# Mitochondrial DNA phylogeography of the Guizhou odorous frog: limited population genetic structure and evidence for recent population size expansion

**DOI:** 10.1080/23802359.2021.1914215

**Published:** 2021-05-04

**Authors:** Shize Li, Gang Wei, Jing Liu, Houqiang Xu

**Affiliations:** a Laboratory of Animal Genetics, Breeding and Reproduction in the Plateau Mountainous Region, Ministry of Education, Collaborative Innovation Center for Mountain Ecology and Agro-Bioengineering (CICMEAB), College of Life Sciences, Guizhou University, Guiyang, China; b Key Laboratory of Animal Genetics Breeding of Guizhou Province, Guizhou University, Guiyang, China; c Department of Food Science and Engineering, Moutai Institute, Renhuai, China; d Biodiversity Conservation Key Laboratory, Guiyang College, Guiyang, China

**Keywords:** Genetic diversity, mitochondrial DNA, *Odorrana kweichowensis*, recent expansion

## Abstract

The Guizhou odorous frog *Odorrana kweichowensis* is endemic to Guizhou Province, China. In this study, a comparative analysis of the mitochondrial COI and ND2 gene sequences was performed to examine genetic diversity in 109 individuals from ten localities across the geographic range of the species. Haplotype diversity and nucleotide diversity were 0.576 and 0.00055, respectively. Phylogenetic analyses almost nested all haplotypes into one lineage. AMOVA indicated that total variation was mainly derived from variation within individual populations. Neutral tests indicated that a recent expansion occurred in the total population. *F*st estimations indicated that genetic divergence was not correlated with geographic distance. Accordingly, the species probably experienced a recent population expansion, and there no obvious population genetic structure is apparent. The findings provide useful information for the conservation of this species.

## Introduction

The odorous frog *Odorrana kweichowensis* Li et al. 2018 (Amphibia, Anura, Ranidae) was recently described to science from mountainous streams in Guizhou Province, China (Li et al. 2018). Although Li et al. (2018) reported three populations of *O. kweichowensis* in the northern part of Guizhou Province, China, the species was suggested to occur in parapatry with its phylogenetically closest species, *O. schmackeri,* and to have a wider distributional range in Guizhou Province and perhaps into northern Guiangxi Province, China (Li et al. 2015, 2018; Zhu 2016). The karst and mountainous topography (e.g., Dalou Mountain and Wuling Mountain series), including some deep valleys (e.g., Wujiang River and Chishui River), in this region would be expected to impede gene flow between populations of amphibians restricted to mountainous streams (e.g., Che et al. 2010; Yan et al. 2013).

We conducted comprehensive surveys of *O. kweichowensis* and collected a series of specimens from the northern part to the southern edge of Guizhou Province, China. These surveys showed that a variety of human-caused threats, such as habitat destruction, overharvesting, and pollution, have likely caused a recent decline in the number of *O. kweichowensis*, as also reported in Li et al. (2018). Based on this information, the species was classified as Vulnerable in the IUCN Red List of Threatened Species (IUCN 2021). Understanding population history and genetic diversity is fundamental for conserving species (reference), but until now there has been limited attention toward *O. kweichowensis*.

Mitochondrial DNA (mtDNA) markers are frequently used for inferring levels of population genetic divergence and structure (Liu et al. 2015; Wang et al. 2017) due to their rapid rates of evolution and maternal inheritance (Sun et al. 2012). In this study, the mitochondrial Cytochrome c oxidase subunit I (COI) and NADH dehydrogenase subunit 2 (ND2) genes were used to reveal the population genetic structure and diversification history of *O. kweichowensis* to provide information that will assist in the conservation of the species.

## Materials and methods

**Figure 1.**
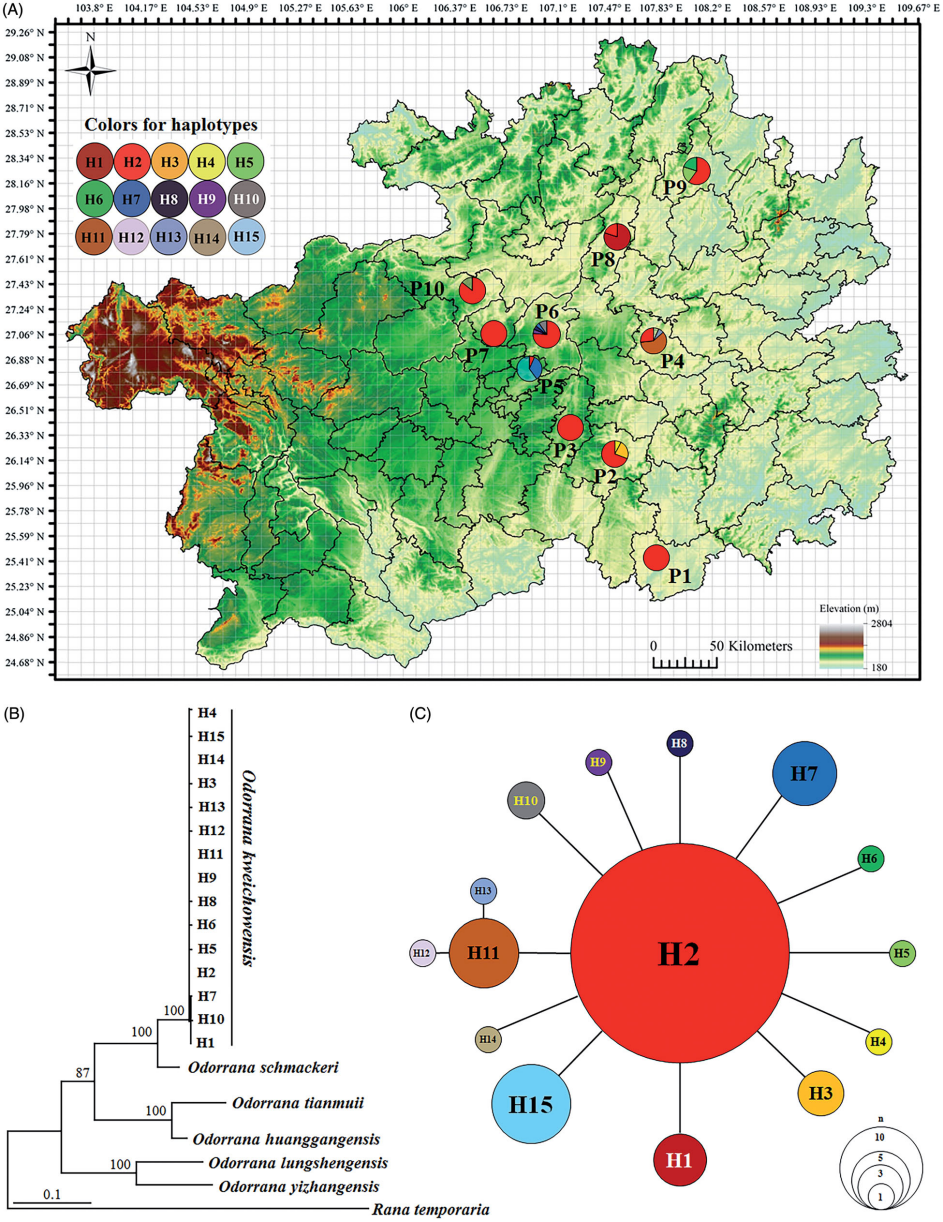
Sampling localities in this study and phylogenetic relationships of *Odorrana kweichowensis*. (A) Sampling localities (P1–P10) in this study. (B) Maximum likelihood tree based on COI + ND2 gene sequences of haplotypes of *O. kweichowensis*. Bootstrap supports with >50% values are denoted near nodes. (C) Haplotype network of *O. kweichowensis*. The circle size is proportional to the number of samples.

A total of 109 specimens were collected from ten localities (P1–P10) in Guizhou Province, China that span the known geographical range of *O. kweichowensis* (Figure 1(A); Table 1). The Animal Care and Use Committee of Guiyang College provided full approval for this study (Number: GYU2018040002). Field work was approved by the Management Office of the Kuankuoshui Nature Reserve (project number: KKS201504003). Specimens were euthanized before taking muscle tissue. Muscle tissue samples were taken and preserved separately in 99% ethanol prior to fixation of the voucher specimen in 10% formalin. Preserved specimens were deposited in the Moutai Institute (voucher numbers in Table 1). Total DNA was extracted using a standard phenol-chloroform extraction protocol (Sambrook et al. 1989). Two fragments of the mitochondrial COI and ND2 genes were amplified. For COI, Chmf4 (5′-TYTCWACWAAYCAYAAAGAYATCGG-3′) and Chmr4 (5′-ACYTCRGGRTGRCCRAARAATCA-3′) were used following Che et al. (2012) and for ND2, Ile-LND2(ATAGGGAGACTTATAGGGGTTC) and Asn-HDN2 (CTAAGTCATTACGGGATCGAGGCC) were used following Li et al. (2015). Gene fragments were amplified under the following conditions: an initial denaturing step at 95 °C for 4 min; 36 cycles of denaturing at 95 °C for 30 s, annealing at 46 °C (for COI)/57 °C (for ND2) for 40 s and extending at 72 °C for 70 s. Sequencing was conducted using an ABI3730 automated DNA sequencer at Shanghai DNA BioTechnologies Co., Ltd. (Shanghai, China).

**Table 1.** Genetic diversity and neutrality tests of *O. kweichowensis*.

Population ID	Locality	Longitude (°E)	Latitude (°N)	Altitude (m)	*n*	Haplotype (voucher No.)	*h*	*π*	Tajima’s D	Fu’s *F*s
P1	Libo Co., Guizhou Prov.	108.070361	25.288306	512	15	H2 (ML20190802023-37)	0.000	0.00000	/	/
P2	Duyun City, Guizhou Prov.	107.376092	26.359197	1055	13	H2 (DY20180428002, DY20180428004, DY20180428016, DY20180618001-04, DY20180618006-07), H3 (DY20180428003, DY20180618005, DY20180618008), H4 (DY20180428005)	0.500	0.00041	−0.46216	−0.413
P3	Guiding Co., Guizhou Prov.	107.239431	26.346853	1078	7	H2 (GD20200726001-07)	0.000	0.00000	/	/
P4	Huangping Co., Guizhou Prov.	107.644306	26.942942	879	13	H2 (HP20200726006, HP20200726008,HP20200726010-11), H11 (HP20200726001-02, HP20200726005, HP20200726007,HP20200726009, HP20200726012-13 ), H12 (HP20200726003), H13 (HP20200726004)	0.654	0.00059	−0.64598	−1.079
P5	Guiyang City, Guizhou Prov.	106.910961	26.78615	1111	15	H2 (GY20190803002), H7 (WD20200814005, WD20200725002-03, GY20190803003-04), H15(WD20200814001-03, WD20200725005-08, WD20200725011-12)	0.562	0.00076	1.60586	0.907
P6	Kaiyang Co., Guizhou Prov.	107.032447	26.909936	978	21	H2 (KY20190804001-06, KY20190804008-14, KY20200724003-04, KY20200724006), H7 (KY20190804007), H8 (KY20190804016), H9 (KY20200724001), H10 (KY20200724002, KY20200724005)	0.424	0.00036	−1.65358	−3.127*
P7	Xifeng Co., Guizhou Prov.	106.846331	27.204672	720	8	H2 (XF20200723001-08)	0.000	0.00000	/	/
P8	Meitan Co., Guizhou Prov.	107.528164	28.146197	947	5	H1 (GYU20130917001,GYU20130917003-05), H2 (GYU20130917002)	0.400	0.00031	−0.8165	0.090
P9	Dejiang Co., Guizhou Prov.	107.985469	28.318511	1114	5	H2 (DJ20160530005, DJ20160531001, DJ20160531004), H5 (DJ20160530006), H6 (DJ20160530007)	0.700	0.00061	−0.97256	−0.829
P10	Jinsha Co., Guizhou Prov.	27.538753	106.00008	939	7	H2 (JS20150803008,JS20150804004-05, JS20200720001-03), H14 (JS20150803005)	0.286	0.00022	−1.00623	−0.095
Total	/	/	/	/	109	H1-H15	0.576	0.00055	−1.97196**	−13.834**

*n*: number of samples; *h*: haplotype diversity; *π*: nucleotide diversity. Significance level: *p < 0.05, **p < 0.01.

Sequences were assembled and aligned using the ClustalW module in BioEdit v.7.0.9.0 (Hall 1999) under the default settings. The two gene fragments were translated to amino acid sequences in MEGA 6.06 (Tamura et al. 2013), adjusted for open reading frames, and checked to ensure absence of premature stop codons. No-sequenced fragments were treated as missing data. All haplotype sequences were submitted to GenBank under accession numbers MW067106–MW067120 for COI and MW071122–MW071136 for ND2. The two gene fragments were concatenated into a single fragment for each individual for analyses.

Variable sites, conserved sites, and nucleotide composition were estimated using MEGA 6.06. Haplotype diversity (*h*) and nucleotide diversity (*π*) were estimated using DnaSP v.5 (Librado and Rozas 2009). Genetic signals of departure from neutrality or potential population expansion were estimated for populations using Tajima’s D (Tajima 1989) and Fu’ *F*s (Fu 1997) statistics, estimated in DnaSP. Pairwise uncorrected *p*-distances between populations were estimated using MEGA 6.06. The hierarchical distribution of overall diversity was determined using an Analysis of Molecular Variation (AMOVA), as implemented in Arlequin 3.11 (Excoffier et al. 2005) and *F*-statistics (*F*st) among the populations was estimated in DnaSP. For the phylogenetic analyses based on COI + ND2 genes, corresponding sequences of one *O. schmackeri*, one *O. tianmuii*, one *O. huanggangensis,* one *O. yizhangensis*, one *O. lungshengensis,* and one *Rana temporaria* were downloaded from GenBank (GenBank accession Nos.: MH193609, KX233866, MH193595, MH193616, MH193608 and MH536744) and aligned to the *O. kweichowensis* dataset as described above. Phylogenetic relationships were reconstructed using Maximum Likelihood (ML) as implemented in the program PHYML v. 3.0 (Guindon et al. 2010). For ML analyses, we ran JMODELTEST v. 2.1.2 (Darriba et al. 2012) with Akaike information criteria on the alignment, resulting in the best-fitting nucleotide substitution models of GTR + I for the data used in ML. For the ML analysis, branch supports were drawn from 10,000 nonparametric bootstrap replicates. Finally, haplotype networks were constructed using the maximum parsimony method in TCS v. 1.21 (Clement et al. 2000).

## Results

The aligned sequence matrix of COI + ND2 contained 1311 bp (COI with 528 bp and ND2 with 783 bp). The proportions of base types were A = 27.2%, T = 27.8%, C = 31.8%, and G = 13.1%, with (A + T) = 55.0% significantly higher than (C + G) = 44.9%. Fifteen haplotypes were found among the 109 individuals of ten populations of *O. kweichowensis* (Table 1; Figure 1). Haplotype H2 was shared by all populations, haplotype H7 was shared by two populations (P5 and P6), and all other haplotypes were unique to each population (Figure 1(A); Table 1). Five haplotypes were observed in P6 (H2, H7, H8, H9, and H10), four haplotypes were observed in P4 (H2, H11, H12, and H13), three haplotypes were observed in P2 (H2, H3, and H4) and P9 (H2, H5, and H6), two haplotypes were observed in P8 (H1 and H2) and P10 (H2 and H14), and only one was found in each of the remaining three populations (Figure 1(A); Table 1).

The total haplotype diversity of all populations was moderate with 0.577, and haplotype diversity was moderate or low in each population (Table 1). P9 showed the highest haplotype diversity (0.700), followed by P4 (0.654), P5 (0.562), then P2 (0.500), P6 (0.424), P8 (0.400) and P10 (0.268), and the other seven populations exhibited only one haplotype. The total nucleotide diversity of the total population was 0.00055. P5 showed the highest nucleotide diversity (0.00076), followed by P9 (0.00061), P4 (0.00059), P2 (0.00041), P6 (0.00036), P8 (0.00031) and P10 (0.00022), and the other three populations exhibited only one haplotype.

In the total population, Tajima’s D values and Fu’s *F*s values were significantly negative (*p* < 0.01; Table 1), suggesting a recent population expansion. P2, P4, P6, P9 and P10 showed a negative Tajima’s D value with no significance and P8 showed a positive Tajima’s D value with no significance. In Fu’s *F*s tests, P2, P4, P9 and P10 showed negative values with no significance, P6 showed a negative value with significance, and P5 and P8 showed positive values with no significance (Table 1).

Pairwise genetic distances among the populations ranged from 0.1% to 0.2% with an average value of 0.2%. AMOVA showed that only 37.4% of the molecular variance was attributed to differentiation among populations, whereas 62.6% of the molecular variance was derived from within populations. The highest *F*st value among the populations was 0.750, and the lowest was 0.000 (Table 2).

**Table 2.** *F*-statistics (*F*st) among the populations of *O. kweichowensis*.

	P1	P2	P3	P4	P5	P6	P7	P8	P9
P1									
P2	0.125								
P3	0.000	0.125							
P4	0.545	0.433	0.545						
P5	0.469	0.384	0.469	0.506					
P6	0.020	0.079	0.020	0.430	0.361				
P7	0.000	0.125	0.000	0.545	0.469	0.020			
P8	0.750	0.576	0.750	0.645	0.599	0.583	0.750		
P9	0.000	0.054	0.000	0.370	0.329	0.007	0.000	0.500	
P10	0.000	0.085	0.000	0.467	0.407	0.013	0.000	0.636	0.000

In the ML tree (Figure 1(B)), all samples of *O. kweichowensis* clustered into one clade (bootstrap supports, bs = 100) that was recovered as sister to *O. schmackeri*. The haplotype network showed that H2 was occupied by 70 samples, H1–H11, H14 and H15 each independently linked to H2, and H12 and H13 linked to H11 (Figure 1(C)).

## Discussion

The geographical topology within the distributional range of *O. kweichowensis* in southwestern China is complex and has been proposed to present mountain or river barriers for gene flow that promote speciation (Che et al. 2010; Yan et al. 2013). Mitochondrial genes have often been used to investigate population genetic structure within frog species in southwestern China and have often revealed considerable divergence between populations (e.g., Wang et al. 2012, 2013, 2017). However, our results based on the mitochondrial COI and ND2 gene sequences found low genetic diversity and did not find obvious population genetic structure in the frog *O. kweichowensis*. In this study, only 16 haplotypes were found among 109 specimens from 10 populations across the range of the species, and one haplotype (H2) occurred in all populations, indicating that all examined populations of the species might have a recent common origin. The genetic distances between populations was very low (average of 0.2% between samples). Further, AMOVA also suggested that more than 60% molecular variance was attributed to the differentiation within populations rather than between populations. This point was also evidenced by neutrality tests that indicated a significant recent population size expansion in the total population of the species. Obviously, the recent population expansion was mainly derived from the ubiquitous distribution of haplotype H2. However, most populations of the frog have been experienced some minimal divergence, for example, the haplotype network indicated that in P4, haplotypes H12 and H13 were probably derived from H11 rather than the common H2. These results indicate that the frogs of the species have probably experienced shallow population divergences only very recently. Nevertheless, based on current results, we could not deduce the ancestral region or expansion center due to seven populations exhibiting unique haplotypes with low genetic diversity.

In summary, genetic diversity and structure in *O. kweichowensis* were relatively low, indicating that it might be a young species or has experienced bottleneck effects (Miracle and Campton 1995). Future work with more markers might clarify the cause of this pattern of relatively low genetic diversity and structure. However, the existing genetic diversity within this Guizhou-endemic frog species should be protected in light of threats from human activities. Populations P4, P5, P6 and P9 harbor most of the genetic diversity, and so should be the highest priority for conservation.

## Data Availability

The data of this study are openly available in figshare at http://doi.org/10.6084/m9.figshare.13152869. All haplotype sequences were submitted to GenBank under accession numbers MW067106–MW067120 for COI and MW071122–MW071136 for ND2.
